# Active DNA demethylation: mechanism and role in plant development

**DOI:** 10.1007/s00299-017-2215-z

**Published:** 2017-10-12

**Authors:** Yan Li, Suresh Kumar, Weiqiang Qian

**Affiliations:** 1 State Key Laboratory of Protein and Plant Gene Research, Peking-Tsinghua Center for Life Sciences, School of Life Sciences, Peking University, Beijing, 100871 China; 2 Center for Plant Biology, School of Life Sciences, Tsinghua University, Beijing, 100084 China; 3 Division of Biochemistry, ICAR-Indian Agricultural Research Institute, New Delhi, 110012 India

**Keywords:** Demeter, ROS1, Active DNA demethylation, DNA methylation, Plant development

## Abstract

Active DNA demethylation (enzymatic removal of methylated cytosine) regulates many plant developmental processes. In *Arabidopsis*, active DNA demethylation entails the base excision repair pathway initiated by the Repressor of silencing 1/Demeter family of bifunctional DNA glycosylases. In this review, we first present an introduction to the recent advances in our understanding about the mechanisms of active DNA demethylation. We then focus on the role of active DNA demethylation in diverse developmental processes in various plant species, including the regulation of seed development, pollen tube formation, stomatal development, fruit ripening, and nodule development. Finally, we discuss future directions of research in the area of active DNA demethylation.

## Introduction

DNA methylation is an evolutionarily conserved epigenetic mechanism that controls numerous biological processes including gene imprinting, tissue-specific gene expression, inactivation of transposable elements (TEs), paramutation, and stress responses. In plants, DNA methylation occurs in the CG, CHG and CHH contexts (where H represents A, C, or T). The methylation level is dynamically controlled by establishment, maintenance and removal of cytosine methylation (DNA demethylation). The establishment and maintenance of DNA methylation in plants are well understood and have been comprehensively reviewed (Law and Jacobsen 2010; Matzke and Mosher 2014; Movahedi et al. 2015). Here we review DNA demethylation.

DNA demethylation occurs either by passive or active process. Passive DNA demethylation refers to the loss of DNA methylation during DNA replication because of reduction or inactivation of enzymes that contribute to DNA methylation (Zhu 2009). Passive DNA demethylation has been reported during gametophyte development in flowering plants. Male gametophyte generation consists of two- or three-celled pollens that deliver two sperm cells to embryo sac at fertilization. In the pollen, TEs were found to be unexpectedly reactivated only in the vegetative cell, which accompanies the sperm cell but does not provide DNA to the fertilized zygote (Slotkin et al. 2009). In *Arabidopsis*, reduced expression of the RNA-directed DNA methylation (RdDM) pathway components during male gametogenesis results into passive DNA demethylation in the vegetative cell (Slotkin et al. 2009). Similarly, passive DNA demethylation may also occur in the central cell (the companion cell of the egg that develops into endosperm after fertilization) during female gametophyte development. Transcriptional repression of the maintenance DNA methyltransferase MET1 was found to be associated with genome-wide DNA demethylation in the central cell (Jullien et al. 2008). However, results from a recent study argue against decreased MET1 expression in the central cell (Park et al. 2016), making the involvement of passive DNA demethylation in female gametogenesis controversial.

Active DNA demethylation involves the enzymatic removal of methylated cytosine. In plants, this process is initiated by a family of DNA glycosylases including Demeter (DME), Repressor of silencing 1 (ROS1), Demeter-like 2 (DML2), and Demeter-like 3 (DML3). A base excision repair (BER)-dependent mechanism then completes the process (Penterman et al. 2007; Zhu 2009). Active DNA demethylation is not only crucial for genome-wide epigenetic reprogramming but also mediates locus-specific gene activation during plant development (Hsieh et al. 2009). In this paper, we review the recent progress in understanding the mechanisms of active DNA demethylation in plants and highlight the role of this process in plant development.

## Mechanisms of active DNA demethylation in plants

### BER-mediated active DNA demethylation

DME, ROS1, DML2, and DML3 are bifunctional DNA glycosylases involved in BER by hydrolyzing the glycosylic bond between the base and its deoxyribose residue and cleaving the DNA backbone at the abasic site. This removes methylated cytosine irrespective of its sequence context and generate a single-nucleoside gap (Choi et al. 2002; Gong et al. 2002; McCullough et al. 1999; Morales-Ruiz et al. 2006; Ortega-Galisteo et al. 2008). *ROS1, DML2*, and *DML3* are ubiquitously expressed in vegetative tissues and exhibit partial functional redundancy (Ortega-Galisteo et al. 2008; Penterman et al. 2007; Zhu et al. 2007). However, an *Arabidopsis* triple mutant of *ROS1, DML2*, and *DML3* (*rdd*) showed DNA hypermethylation (increased level of methylated cytosine) at nearly 9000 loci, which was a considerably higher number than the number of loci specifically targeted by ROS1 (approximately 5000) (Qian et al. 2012), suggesting that DML2 and DML3 also have unique functions. *DME* is mainly expressed in the central cell (Choi et al. 2002; Park et al. 2016). Transient *DME* expression has also been detected in the vegetative cell (Park et al. 2017; Schoft et al. 2011). In the central and the vegetative cells, DME preferentially targets short, AT-rich and nucleosome-free euchromatic TEs (Ibarra et al. 2012).

**Fig. 1:**
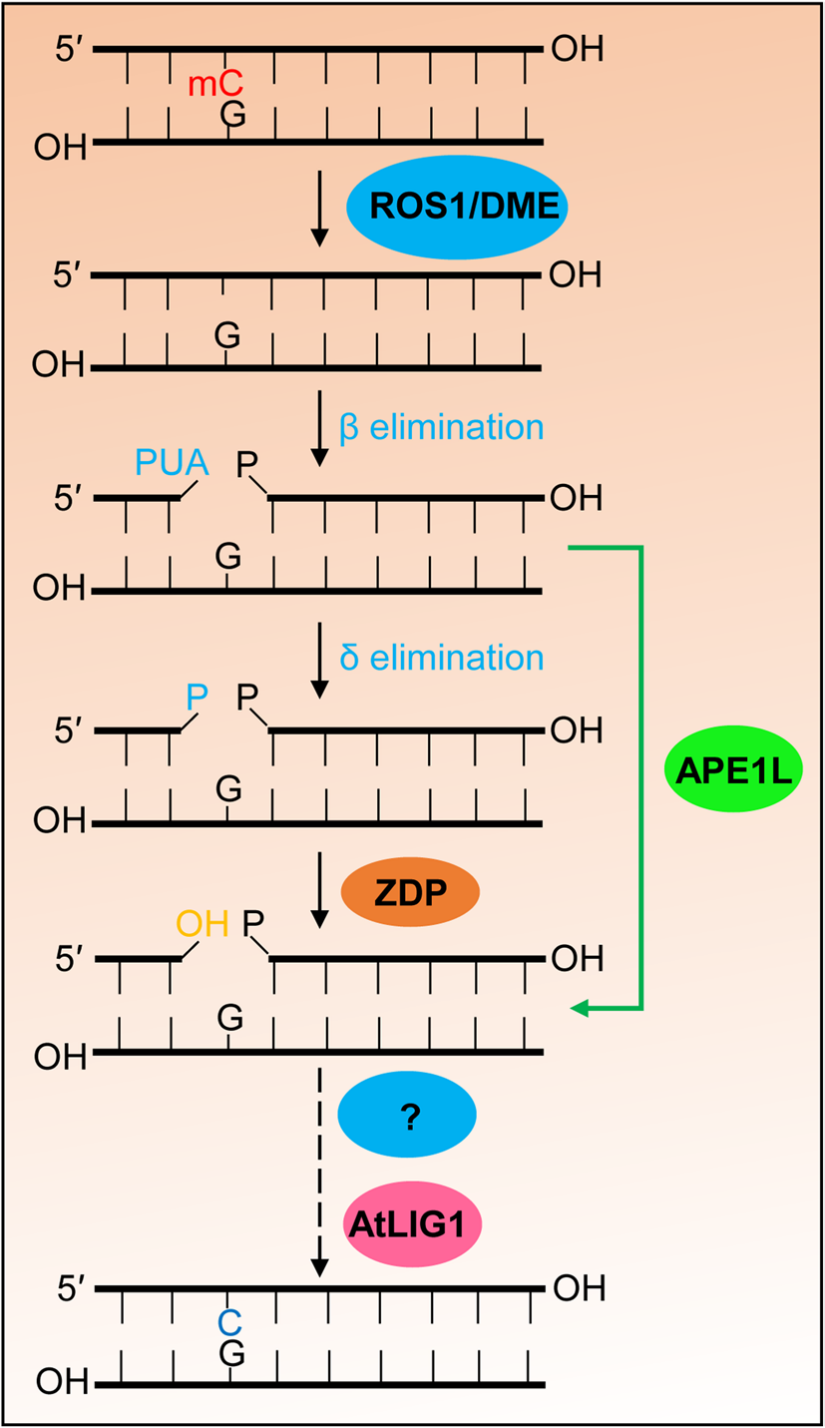
Base excision repair (BER)-mediated active DNA demethylation in plants. ROS1 and DME are bifunctional DNA glycosylases that remove 5-methylcytosine (mC) and cleave the DNA backbone at abasic site via β or β, δ elimination reaction(s), generating a gap with 3′-PUA or 3′-phosphate terminus. 3′-PUA and 3′-phosphate are converted to 3′-OH by .APE1L and ZDP, respectively. The gap is then filled with an usual (unmethylated) cytosine by the actions of an unknown DNA polymerase and AtLIG1

DME and ROS1 catalyze β elimination reaction or successive β, δ elimination reaction when cleaving the DNA backbone (Fig. 1). The β elimination reaction creates a gap with 3′-phospho-α, β-unsaturated aldehyde (3′-PUA), while β, δ elimination reaction creates a gap with a 3′-phosphate terminal (Agius et al. 2006; Lee et al. 2014; Morales-Ruiz et al. 2006). Both 3′-phosphate and 3′-PUA must be converted to 3′-hydroxyl (3′-OH) so that DNA polymerase and ligase activities can fill in the gap. Zinc finger DNA 3′-phosphoesterase (ZDP) (homologue of human polynucleotide kinase 3′-phosphatase) converts the 3′-phosphate group to a 3′-OH group (Fig. 1) (Jilani et al. 1999; Martinez-Macias et al. 2012). Among three apurinic/apyrimidinic (AP) endonuclease-like proteins APE1L, APE2, and apurinic endonuclease-redox protein in *Arabidopsis*, APE1L (homologue of human APE1) can potently process 3′-PUA to generate 3′-OH (Fig. 1) (Lee et al. 2014; Li et al. 2015b). ZDP-mediated reaction and APE1L-mediated reaction comprise two branches of the DNA demethylation pathway downstream of ROS1 and DME. Both ZDP and APE1L colocalize and interact with ROS1. ZDP and APE1L dysfunction was reported to cause DNA hypermethylation at approximately 1500 and 3500 endogenous loci, respectively (Li et al. 2015b). *ZDP* is expressed in both vegetative and reproductive tissues (Martinez-Macias et al. 2012), whereas *APE1L* is mostly expressed in siliques (Lee et al. 2014). Unlike *ZDP* mutation, which has a slightly greater effect on TE regions, *APE1L* mutation preferentially causes DNA hypermethylation at genic regions (Li et al. 2015b). While the DNA polymerase responsible for active DNA demethylation remains unknown, the DNA ligase, that creates a phosphodiester bond and joins the two ends of DNA strands after filling the gap with an unmethylated cytosine nucleotide, was identified to be AtLIG1 (Fig. 1) (Andreuzza et al. 2010; Li et al. 2015c).

### Regulation of DNA glycosylases

**Fig. 2:**
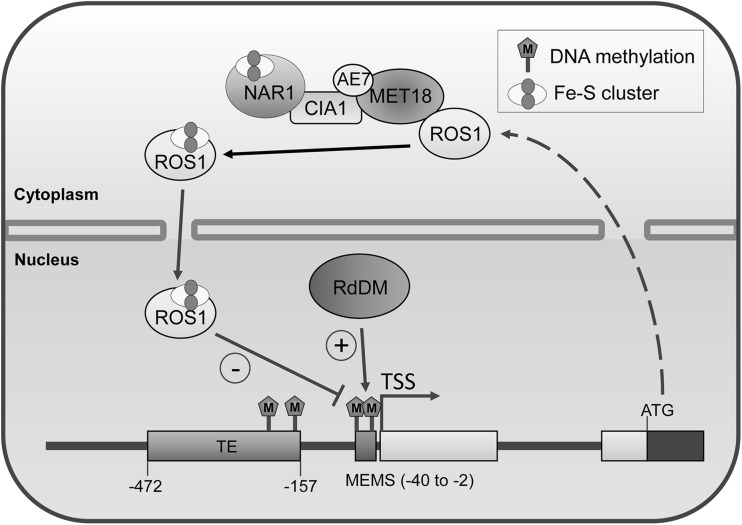
Regulation of ROS1 by the cytosolic iron-sulfur cluster assembly (CIA) pathway and DNA methylation. In the cytoplasm, the CIA pathway component MET18 directly interacts with ROS1, and helps transferring the Fe–S cluster to ROS1, which then gets transported into the nucleus to perform active DNA demethylation. The DNA methylation level at the MEMS region in *ROS1* promoter is tightly controlled by ROS1 and RdDM, and higher DNA methylation level promotes *ROS1* expression. (+) and (−) symbols represent a positive and negative effect on *ROS1* expression, respectively

*ROS1* transcript level is tightly controlled by the methylation level of an RdDM target sequence (DNA methylation monitoring sequence, MEMS) that lies between *ROS1* 5′ UTR and the promoter TE region (from − 40 to − 2 bp upstream of the *ROS1* transcriptional start site) (Lei et al. 2015) (Fig. 2). When cellular RdDM activities are high or DNA demethylation activities are low, the methylation level of MEMS increases. Increased DNA methylation at MEMS promotes *ROS1* expression and active DNA demethylation (Lei et al. 2015). By contrast, in RdDM mutants or when DNA demethylation activities are high, the methylation level at MEMS is low and *ROS1* expression is suppressed. DNA methylation at MEMS promotes *ROS1* expression likely through attracting transcriptional activators (Lei et al. 2015). The methylation-sensitive expression of *ROS1* helps in maintaining DNA methylation/demethylation homeostasis (Williams et al. 2015). A recent study comprehensively analyzed the necessity and sufficiency of different *DME* genomic regions for tissue-specific *DME* expression (Park et al. 2017). The minimal promoter sequence that can drive *DME* expression in the central cell was identified to lie within the *DME* transcriptional unit, from + 202 to + 559 bp downstream of the *DME* transcriptional start site (Park et al. 2017). Importantly, the sequences required for the central cell- and vegetative cell-specific *DME* expression were narrowed down to a 15-bp region (from + 448 to + 462 bp) and a 47-bp region (from + 416 to + 462 bp), respectively. The homeodomain-leucine zipper family of transcription factors was predicted to bind the overlapping part of these two regions and thereby regulate *DME* expression.

ROS1 enzymatic activity was demonstrated to be regulated by the cytosolic iron–sulfur cluster assembly (CIA) pathway components asymmetric leaves1/2 enhancer 7 (AE7) and METhionine requiring18 (MET18) (Duan et al. 2015; Luo et al. 2012; Wang et al. 2016). MET18 can directly interact with ROS1 in the cytoplasm (Fig. 2). MET18 dysfunction causes compromised ROS1 enzymatic activity and DNA hypermethylation at ROS1 target loci (Duan et al. 2015; Wang et al. 2016). Similar to ROS1, DME is an iron–sulfur cluster binding protein and the iron–sulfur binding motif is crucial for its enzymatic activity (Mok et al. 2010). Although AE7 and MET18 may not regulate DME activity, the other two CIA pathway components, NAR1 and DRE2, regulate the expression of *FWA*, a DME target gene, indicating the involvement of the iron–sulfur cluster assembly in DME-mediated active DNA demethylation (Buzas et al. 2014; Nakamura et al. 2013).

### Targeting of the active DNA demethylation machinery

ROS3 was reported to guide ROS1 to a subset of its target loci by associating with small RNAs (Zheng et al. 2008). Other studies reported that histone acetyltransferase increased DNA methylation1 (IDM1), also called ROS4, plays a role in ROS1 targeting. IDM1 recognizes loci with high CG methylation but low histone H3K4 dimethylation or trimethylation and adds acetyl marks on H3K18 and H3K23 at these loci. Thus, by creating a permissive chromatin state, IDM1 facilitates ROS1 targeting (Li et al. 2012; Qian et al. 2012). Soon after the discovery of IDM1, IDM2 (also called ROS5), IDM3 (also called increased DNA methylation2-like1, IDL1), methyl-CpG-binding protein 7 (MBD7), and Harbinger transposon-derived protein1 (HDP1) and HDP2, which form a complex with IDM1, were identified (Duan et al. 2017; Lang et al. 2015; Li et al. 2015a; Qian et al. 2014; Zhao et al. 2014). MBD7 and HDP2 bind DNA and jointly ensure that IDM1 is targeted to the regions with high CG methylation. IDM2 and IDM3 likely play a role in connecting IDM1 and MBD7, and HDP1 probably mediates the interaction between IDM1 and HDP2. Meanwhile, IDM2 and IDM3 may serve as molecular chaperones that regulate IDM1 enzymatic activity. Taken together, IDM1-mediated ROS1 targeting involves the collective action of all components of the IDM1 histone acetyltransferase complex. Similar to ROS1 targeting, DME targeting may be regulated by multiple factors. Histone H1 was found to interact with DME and be required for the expression of DME target genes (Rea et al. 2012). SSRP1, a component of the FACT (facilitates chromatin transcription) complex, was also reported to be indispensable for the expression of DME target genes (Ikeda et al. 2011). Because histone H1 and SSRP1 are chromatin-binding proteins, their association with chromatin may control DME targeting. However, the direct evidence for histone H1- and SSRP1-mediated regulation of DME targeting remains to be gathered.

## Role of active DNA demethylation in plant development

The elucidation of mechanisms for active DNA demethylation in plants provides more opportunity to explore the function of active DNA demethylation in gene regulation and plant development. In the last two decades, accumulating evidence indicates that active DNA demethylation regulates diverse biological processes of various plant species (Table 1).

**Table 1:** Functions of the known DNA demethylases in plant development

Demethylase	Plant	Target gene	Function	References
ROS1	*Arabidopsis*	*EPF2*	Stomatal development	Yamamuro et al. (2014)
ROS1, DML2, DML3	*Arabidopsis*	*APOLO*	Auxin-controlled development	Ariel et al. (2014)
DME	*Arabidopsis*	*MEA, FIS2, FWA*	Gene imprinting and embryo development	Choi et al. (2002) Gehring et al. (2006)
DME	*Arabidopsis*	unknown	Pollen germination	Schoft et al. (2011)
TaDME	Wheat	Gliadins, *LMWg*s	Gluten abundance	Wen et al. (2012)
ROS1a	Rice	unknown	Endosperm and embryo development	Ono et al. (2012)
MtDME	Medicago	*NCR*s	Nodule differentiation	Satge et al. (2016)
SlDML2	Tomato	*RIN, NOR, PSY1*	Fruit ripening	Liu et al. (2015) Lang et al. (2017)

### Activation of maternally imprinted genes and regulation of seed development

The term ‘imprinted genes’ refers to genes that are preferentially expressed either from maternally or paternally inherited alleles. Since the report of the first imprinted maize *R* gene, dozens of plant imprinted genes have been identified in the past 20 years (Rodrigues and Zilberman 2015). In *Arabidopsis*, the well characterized maternally imprinted genes (maternally expressed genes, MEGs) include *flowering wa-geningen* (*FWA*) (Kinoshita et al. 2004), MEDEA (MEA) (Gehring et al. 2006), fertilization independent seed 2 (FIS2) (Jullien et al. 2006), maternally expressed PAB C-terminal (MPC) (Tiwari et al. 2008), *AtFH5* (Fitz Gerald et al. 2009), *Agamous-like36* (*AGL36*) (Shirzadi et al. 2011), and *NUWA* (He et al. 2017). These genes are maintained in a default silenced state owing to DNA methylation and repressive histone modifications. In the central cell of the female gametophyte, the maternal alleles of these MEGs are activated by DME-mediated active DNA demethylation. Although DME expression is confined to the central cell and does not persist in the endosperm, maternal hypomethylation (reduced level of methylated cytosine) and activation of these MEGs are epigenetically maintained in the endosperm (Choi et al. 2002). Mutations in the maternal *DME* result in failure of MEG activation and early seed abortion, with endosperm enlarged and embryo growth arrested. Plants homozygous for the *DME*-null mutation do not survive, whereas heterozygous *DME*/*dme-1* and *DME*/*dme-2* plants exhibit a 50% seed abortion phenotype due to the maternal effect (Choi et al. 2002). Notably, not only DME, but also proteins acting downstream of DME, including ZDP, APE1L, and DNA ligase I, have been reported to be essential for proper seed development. Although a single *APE1L* or *ZDP* mutant does not display any abnormal phenotype, the double mutant of *APE1L* and *ZDP* exhibits an embryonic lethal phenotype and the *ape1l* ^*+/−*^ *zdp* ^*−/−*^ plant, when self-pollinated or pollinated with wild-type pollen, produces approximately 50% aborted seeds. Endosperms dissected from the aborted seeds (endosperm of the *ape1l* ^*−/−*^ *zdp* ^*−/−*^ genotype) show DNA hypermethylation of the promoters of selected MEGs, such as *FWA* and *FIS2*, and defective activation of *FWA* and *MEA* (Li et al. 2015b). Characterization of the mutants for DNA ligase I revealed that DNA ligase I exerts a similar maternal effect on MEG activation and seed development (Li et al. 2015c). Besides enzymes directly involved in active DNA demethylation, SSRP1, a component of the FACT complex required for active DNA demethylation in the central cell, also induces a maternal effect on MEG expression and seed development (Ikeda et al. 2011).

Hypomethylation of the maternal genome in the endosperm and activation of MEGs have also been reported in monocots such as rice (Luo et al. 2011; Rodrigues et al. 2013; Zemach et al. 2010) and maize (Waters et al. 2011; Zhang et al. 2011). Thus, active DNA demethylation-dependent gene imprinting probably represents a conserved feature of flowering plants. Although the DME homologue is not evident in rice, one of the four *ROS1* homologues, *ROS1a*, appears to be the functional equivalent of *Arabidopsis DME* (Ono et al. 2012). A mutation in maternal *ROS1a* resulted into arrested endosperm development and deficient embryo development (Ono et al. 2012). A *DME*-like gene was recently identified in maize. Notably, its expression level in the endosperm was higher than that observed in the embryo (Wang et al. 2015). Such elevated expression of the DME-like gene could account for hypomethylation of the maternal genome and activation of MEGs in maize endosperm.

### Regulation of seed storage protein expression

Gliadins, low-molecular-weight glutenin subunits (LMWgs), and high-molecular-weight glutenin subunits (HMWgs) are seed storage proteins that accumulate in wheat and barley endosperms. They constitute the primary source of plant-based proteins in our diets. A recent study revealed that wheat *DME* (*TaDME*) is specifically required for the expression of gliadins and LMWgs, because RNAi suppression of *DME* resulted in the reduction or elimination of specific gliadins and LMWgs, but not HMWgs (Wen et al. 2012). Similarly, demethylation of the promoters of the genes encoding gliadins and LMWgs in barley is necessary for the accumulation of gliadins and LMWgs (Gil-Humanes et al. 2010; Van Herpen et al. 2008), but regulation of HMWgs expression was found to be independent of DNA demethylation (Bethune and Khosla 2012). In light of the difference between the regulation of gliadin and LMWgs expression and that of HMWgs expression in wheat and barley, *TaDME* and *HvDME* suppression is considered a potential strategy for eliminating gliadins and LMWgs that cannot be tolerated by people with celiac disease, while retaining HMWgs that are required for good baking quality (Wen et al. 2012).

### Regulation of pollen tube formation

*Arabidopsis* seeds carrying a paternal mutant *dme* allele develop properly, which suggests that DME dysfunction does not lead to defective sperm fertility (Choi et al. 2002). However, DME does function in the male gametophyte because *DME* expression in the vegetative cell was found to be required for DNA demethylation of *MEA* and *FWA* as well as of the transposon *Mu1a*. A *DME* mutation resulted in impaired vegetative cell germination and pollen tube formation (Schoft et al. 2011). As a result of defective pollen tube formation, self-pollinated *DME*/*dme-1* and *DME*/*dme-2* plants (in the Col-gl background) produce significantly fewer viable heterozygous *DME*/*dme* F1 progenies (approximately 15%) than wild-type progenies (Xiao et al. 2003), otherwise the numbers of two types of progenies should have been equal. Notably, the *ape1l* ^*+/−*^ *zdp* ^*−/−*^ plants also displayed paternal defects such as defective pollen development and germination (Li et al. 2015b), further supporting that active DNA demethylation plays a crucial role in male gametogenesis.

### Regulation of stomatal development

Although no obvious developmental phenotypes were observed in the *ros1* and *rdd* mutants, careful examination of the cellar pattern in the leaf epidermis of these mutants revealed that the *ROS1* mutation leads to a ‘small-cell-cluster’ phenotype (Yamamuro et al. 2014), similar to that seen in the mutants for *epidermal patterning factor2* (*EPF2*), a negative regulator of stomatal development (Hara et al. 2009; Hunt and Gray 2009). The clustered small cells were demonstrated to be stomatal lineage cells. Searching for the methylated region in the *EPF2* promoter and comparison of DNA methylation levels in different mutants further revealed that ROS1 is required for demethylation of a TE in the *EPF2* promoter and *EPF2* expression (Yamamuro et al. 2014). EPF2 negatively regulates *s*peechless (*SPCH*), a factor necessary for asymmetric cell division in stomatal development (Jewaria et al. 2013). When *ROS1* mutates, *EPF2* expression is reduced and *SPCH* expression is depressed. Consequently, stomatal lineage cells are overproduced, with each cell being smaller in size (Yamamuro et al. 2014). These findings suggest that ROS1 mediates lineage-specific DNA demethylation.

### Regulation of fruit ripening

A decrease in the global DNA methylation level in tomato pericarps during ripening suggests the involvement of DNA demethylation in fruit ripening (Teyssier et al. 2008). Passive DNA demethylation unlikely contributes substantially to the DNA demethylation process because limited DNA replication occurs at this developmental stage. Thus, active DNA demethylation may account for the decrease in the DNA methylation levels in tomato pericarps. In support of this, DNA methylation was reported to be specifically removed from 52,095 differentially methylated regions (representing 1% of the tomato genome) when tomato fruit ripens (Zhong et al. 2013). Among the four putative DNA glycosylase genes [DEMETER-like DNA demethylases (DMLs) of *Solanum lycopersicum*] *SlDML1, SlDML2, SlDML3*, and *SlDML4*, only *SlDML2* was reported to show increased expression during tomato fruit ripening. *SlDML2* knockdown using RNAi or *SlDML2* knockout using the CRISPR/Cas9 system was reported to be associated with hypermethylation and repression of the selected ripening genes, which resulted in inhibition of fruit ripening (Lang et al. 2017; Liu et al. 2015). Therefore, Liu et al. (2015) and Lang et al. (2017) suggested that *SlDML2*, a *ROS1* orthologue, is the major DNA glycosylase that regulates tomato fruit ripening. Zhong et al. (2013) identified promoters of more than 200 ripening genes, which could be SlDML2 targets, because these promoters show loss of DNA demethylation during tomato ripening; while Lang et al. (2017) identified 605 genes that are hypermethylated and fail to be up-regulated in the *sldml2* mutants. Notably, the binding site for RIPENING INHIBITOR (RIN), a MADS-box transcription factor, was found in the promoters of ripening genes, and those promoters in the *rin* mutant exhibited DNA hypermethylation (Zhong et al. 2013). These findings suggest that SlDML2-mediated active DNA demethylation is somehow facilitated by RIN. Furthermore, the extent of active DNA demethylation was suggested to be dependent on the *SlDML2* expression level, which is feedback regulated by the ripening genes (Liu et al. 2015). In wild-type fruits, activation of the ripening genes might stimulate *SlDML2* expression, thus leading to thorough DNA demethylation. However, in the fruits of mutants for ripening transcription factors, *SlDML2* induction is blocked, thus resulting in limited DNA demethylation. Unexpectedly, SlDML2-mediated active DNA demethylation was reported to be critical for the transcriptional repression of genes that may become useless after fruit ripening (Lang et al. 2017). Thus, active DNA demethylation seems to play a broader role in the regulation of gene expression than was previously recognized.

### Regulation of nodule development

*Medicago truncatula* is one of the plant species that develop root nodules because of their association with the symbiotic nitrogen-fixing bacteria. A recent study revealed that, among four putative *M. truncatula* DNA glycosylase genes *MtDME, MtDML1, MtROS*, and *MtROSL1, MtDME* is strongly induced in the nodule differentiation zone of *M. truncatula*. Knockdown of *MtDME* resulted in morphological and functional alterations of the *M. truncatula* nodule, such as reduction in nodule size, decrease in plant and bacterial endoreduplication levels and failure to fix nitrogen (Satge et al. 2016). RNA-seq and whole-genome bisulfite sequencing analyses revealed that MtDME is required for demethylation and activation of TEs and genes essential for nodule development, particularly genes coding for nodule-specific cysteine-rich (NCR) peptides that orchestrate differentiation of symbiotic bacteria into nitrogen-fixing bacteroids in the symbiosome (Satge et al. 2016). However, the counterparts of the maternally imprinted genes *MEA, FWA, FIS2*, and *MPC* are not regulated by *MtDME* in the *M. truncatula* nodules. Interestingly, TE activation in the differentiation zone was suggested to result in the production of siRNAs that reinforce DNA methylation in the nodule meristem, similar to siRNAs produced in the companion cells of *A. thaliana* (Calarco et al. 2012; Hsieh et al. 2009; Ibarra et al. 2012).

### Concluding remarks and future perspectives

Recent years have witnessed considerable progress in our understanding of active DNA demethylation. Many of the proteins involved in downstream steps of active DNA demethylation in *Arabidopsis* have been identified. The factors that regulate targeting or enzymatic activation of DNA glycosylases were discovered, and active DNA demethylation was established to play a crucial role in a broad range of developmental processes in several plant species. However, many areas remain unexplored. First, the mechanism(s) for recruitment of DNA glycosylases to specific loci remains unclear. The IDM1 complex only directs ROS1 targeting to a subset of its target loci. Unlike dysfunction of ROS1 or DME, dysfunction of the IDM1 complex components does not give rise to any obvious phenotype (Qian et al. 2012). Thus, there must be unidentified factors that also contribute to ROS1 or DME targeting. Second, few factors that regulate active DNA demethylation activities during specific developmental events have been identified. For instance, auxin has been shown to trigger active DNA demethylation at the *APOLO* (AUXIN REGULATED PROMOTER LOOP RNA) locus in *Arabidopsis* (Ariel et al. 2014). However, factors that connect auxin to active DNA demethylation at the *APOLO* locus remain unknown. Moreover, it is still unclear that how *SIDML2* and *MtDME* are specifically induced during fruit ripening and nodule development, respectively. Identification of key components involved in these processes, for instance, specific transcription factors, will deepen our understanding of the role of active DNA demethylation in these development events. Third, so far, the developmental processes that are known to be regulated by active DNA demethylation are limited. It will be interesting to investigate the role of active DNA demethylation in other important developmental processes, such as flowering, sexual reproduction, seed germination and programmed cell death. Now the role of active DNA demethylation in different biological processes in different species can be assessed by applying the CRISPR/Cas9 technology to genes involved in the active DNA demethylation pathway. Finally, controlling gene expression by manipulating DNA methylation and demethylation status of specific sites is a direction for future research. However, site-specific targeting of factors that facilitate active DNA demethylation using enzymatically inactive Cas9 (dead Cas9, dCas9) fusion has only been tested in mammalian cells (Xu et al. 2016). Development and optimization of RNA-guided dCas9 demethylation technology in plants would help understanding the mechanistic aspects of DNA methylation or demethylation, and the practical applicability of epigenetic manipulation in plants. The genome-editing technologies are improving rapidly, and sooner might reach to a point that may enable plant epigenome engineering to be a success. The coming years are likely to realize improved opportunities for comprehensive functional interrogation of epigenetic marks and engineering crop epigenomes towards stable improvement of agriculturally important traits (Springer and Schmitz 2017; Stricker et al. 2017).

#### **Author contribution statement**

YL, SK, and WQ designed and wrote the review.
